# Extensively and multidrug-resistant bacterial strains: case studies of antibiotics resistance

**DOI:** 10.3389/fmicb.2024.1381511

**Published:** 2024-07-04

**Authors:** Bandar Almutairy

**Affiliations:** Department of Pharmacology, College of Pharmacy, Shaqra University, Shaqra, Saudi Arabia

**Keywords:** antimicrobial resistance, case histories, multidrug resistance (MDR), Saudi Arabia, extensively drug-resistant (XDR)

## Abstract

The development of antibiotic resistance compromises the effectiveness of our most effective defenses against bacterial infections, presenting a threat to global health. To date, a large number of research articles exist in the literature describing the case reports associated with extensively drug-resistant (XDR) and multidrug-resistant (MDR) bacterial strains. However, these findings are scattered, making it time-consuming for researchers to locate promising results and there remains a need for a comparative study to compile these case reports from various geographical regions including the Kingdom of Saudi Arabia. Additionally, no study has yet been published that compares the genetic variations and case reports of MDR and XDR strains identified from Saudi Arabia, the Middle East, Central Europe, and Asian countries. This study attempts to provide a comparative analysis of several MDR and XDR case reports from Saudi Arabia alongside other countries. Furthermore, the purpose of this work is to demonstrate the genetic variations in the genes underlying the resistance mechanisms seen in MDR and XDR bacterial strains that have been reported in Saudi Arabia and other countries. To cover the gap, this comprehensive review explores the complex trends in antibiotic resistance and the growing risk posed by superbugs. We provide context on the concerning spread of drug-resistant bacteria by analyzing the fundamental mechanisms of antibiotic resistance and looking into individual case reports. In this article, we compiled various cases and stories associated with XDR and MDR strains from Saudi Arabia and various other countries including China, Egypt, India, Poland, Pakistan, and Taiwan. This review will serve as basis for highlighting the growing threat of MDR, XDR bacterial strains in Saudi Arabia, and poses the urgent need for national action plans, stewardship programs, preventive measures, and novel antibiotics research in the Kingdom.

## 1 Antimicrobial resistance

Antimicrobial resistance (AMR) is defined as the ability of microorganisms to survive and not to be killed or have their growth inhibited by antimicrobial agents (Shah et al., 2023). AMR develops when bacteria modify themselves and result in the futility of the drugs that were used to treat the infections caused by the same bacteria in the past (Murray et al., 2022). Antibiotic overuse promotes superbugs such as multidrug-resitant (MDR) and extensively drug-resistant (XDR) bacterial strains. Because of these superbugs, mortality and morbidity have spiked repeatedly, posing a severe danger to global public health (Bilal et al., 2024). The period between 1930 and 1960 was a bright era in which many antibiotics were developed by researchers and pharmaceutical industries, but that pace did not seem to be maintained afterwards, due to the emergence of resistant pathogens in contrast to research and development of novel antibiotic agents (Aslam et al., 2018). Pathogens gain resistance as a result of genetic evolution. Since AMR represents a complex health issue, it cannot be comprehended fully by a single research. Furthermore, the formation of AMR is caused by organic and natural mechanisms such as natural selection and adaptation of organisms to their environment (Hernando-Amado et al., 2019).

### 1.1 Types of resistance mechanisms

Bacteria may have intrinsic, acquired, or adaptive antibiotic resistance. The resistance displayed as a result of the bacterium’s inherent characteristics is known as intrinsic resistance. For example; the glycopeptide resistance that Gram-negative bacteria display as a result of the outer membrane’s impermeability within the bacterial cell envelope. When a previously susceptible bacterium develops a resistance mechanism through mutation or the acquisition of new genetic material from an external source (horizontal gene transfer), the bacterium is considered to possess acquired resistance (Christaki et al., 2020). Three primary mechanisms can result in horizontal gene transfer: (i) transformation, which happens when free DNA fragments from a dead bacterium enter a recipient bacterium and merge into its chromosome; (ii) transduction, which is carried out by a bacteriophage; and (iii) conjugation, which occurs by direct physical contact between the bacterial cells (Liu et al., 2020). On the other hand, adaptive resistance is characterized as the ability to withstand one or more antibiotics in response to a particular environmental signal (such as stress, growth status, pH, ion concentrations, nutritional factors, or sub-inhibitory antibiotic doses). Adaptive resistance is temporary, as opposed to inherent and acquired resistance. When the stimulus signal disappears, bacteria that have developed adaptive resistance usually return to their original state and become susceptible to antibiotics (Christaki et al., 2020).

### 1.2 Mechanisms of drug resistance

**Figure 1:**
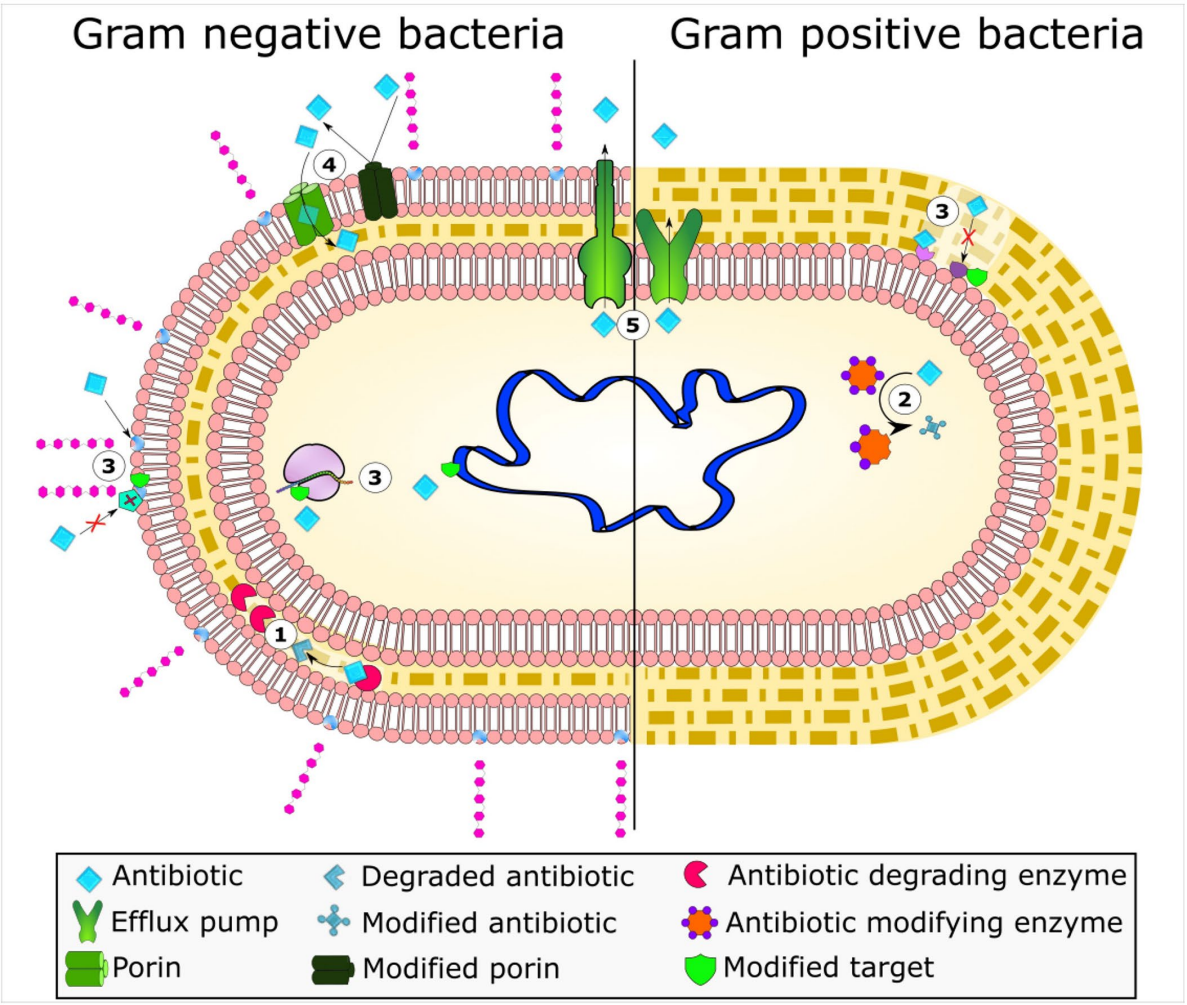
Bacterial mechanisms of resistance to antimicrobial agents. The frequent mechanisms of antibiotic resistance observed in bacteria include enzymatic hydrolysis (1), enzymatic antibiotic modifications through group transfer and redox process (2), alterations to antibiotic targets (3), decreased antibiotic permeability through porin alteration (4), and active antibiotic extrusion via membrane efflux pumps (5). Reprinted from Figure 1 in “Bacterial resistance to antimicrobial agents” by Varela et al. (2021), licensed under Creative Commons Attribution (CC BY) license (https://creativecommons.org/licenses/by/4.0/).

Antimicrobial drugs are used for treating various illnesses, but resistance to these drugs is not a recent development as penicillin resistance evolved quickly after its discovery in 1944 (Agyeman et al., 2022). Bacteria adopt various sophisticated mechanisms of resistance such as the inactivation of antibacterial drugs by enzymes produced by the bacteria itself, efflux pump systems, or reduced permeability, and target site modifications (Figure 1).

#### 1.2.1 Drug efflux pump system

The plasma membrane of bacteria has a specialized protein structure known as efflux pumps. These pumps identify the foreign particles and pump them out, preventing their accumulation inside the bacteria. These pumps have broad antibiotic action, as evidenced by MDR bacteria, or they are substrate-specific. Both Gram-positive and Gram-negative bacteria have efflux pump systems that expel tiny compounds out of bacterial cells (Henderson et al., 2021). The discovery of the first tetracycline efflux mechanism, which is the primary cause of tetracycline resistance, had been identified (Berglund et al., 2020). The five main families of efflux pumps that are now known to exist are the ATP-binding cassette (ABC) families, the main facilitator superfamily (MFS), multidrug and toxic compound extrusion (MATE), resistance-nodulation-division (RND), and small multidrug resistance (SMR). The RND family is not unique only to Gram-negative bacteria, as multiple studies have reported their presence in gram positive strains for instance; Gene yerP, involved in surfactin self-resistance in *Bacillus subtilis* was reported by Tsuge et al. (2001) and *MmpS5/MmpL5* as an efflux pump in Mycobacterium species (Briffotaux et al., 2017). Furthermore, TetR family regulator FarR and efflux pump *FarE*, which give resistance to antimicrobial drugs, are encoded by different genes in *Staphylococcus aureus* (a gram-positive bacterium). The new *TetR* family regulator (TFR) in *S. aureus*, *FarR*, was shown to bind DNA and have sensor specificity, as reported by Alnaseri et al. In contrast to most characterized TFRs, which repress a divergently transcribed gene, Alnaseri et al. discovered that *FarR* is required to enhance the expression of the divergently transcribed *farE* gene, which codes for an RND family efflux pump that is activated in response to antimicrobial unsaturated fatty acids. The fatty acid kinase *FakA*, which catalyzes the initial metabolic step in the incorporation of exogenous unsaturated fatty acids into phospholipid, was necessary for the induction of *farE* (Alnaseri et al., 2019). Lastly, clinical antibiotic resistance is often correlated with efflux pump overexpression (Garcia et al., 2022).

#### 1.2.2 Target modification

Another common mechanism of resistance is modification or mutation in the binding site, where the target is modified and the drug cannot bind to the specific target (Schaenzer and Wright, 2020). Single point mutations may reduce the rifampin binding affinity while preserving polymerase activity, ensuring normal bacterial function. The rifampin target RNA polymerase can be mutated and macrolide binding can be prevented by ribosome methylation. Steric constriction brought about by the methylation interacts primarily with the substituted macrolide backbone (Pelchovich et al., 2013).

#### 1.2.3 Drug metabolism

Antibiotic molecule modification is one of the strategies to produce enzymes that inactivate the drug molecule, enabling the antibiotics to interact with targets (Schaenzer and Wright, 2020). It is an enzyme-based process whereby an active drug molecule is deactivated through an enzyme produced by resistant bacterial cells. This deactivation process includes hydrolysis, group transfer, or redox reactions (Luthra et al., 2018). For example, the beta-lactam ring of penicillin, cephalosporin, and carbapenem is destroyed by hydrolysis through the beta-lactamase enzyme produced by bacteria such as *E. coli*, *K. pneumonia*, and species of the *Enterobacter* genus. Beta-lactamases destroy the amide bond of the B-lactam ring (Glen and Lamont, 2021).

### 1.3 Onset of MDR, XDR strains

Superbug, XDR, and MDR strains have become increasingly common in recent years, raising an alarming situation for healthcare. MDR strains are commonly recognized by their ability to withstand at least one of three or more distinct classes of antibiotics, including ampicillin, sulfonamides (trimethoprim-sulfamethoxazole), and chloramphenicol, however XDR strains are known to be resistant to all but one or two antimicrobials, including ampicillin, sulfonamides (trimethoprim-sulfamethoxazole), chloramphenicol, and fluoroquinolones (ciprofloxacin), as well as third-generation cephalosporins (ceftazidime, cefuroxime, and ceftriaxone), leaving few effective treatment options, such as piperacillin/tazobactam, azithromycin, and carbapenem (Fodor et al., 2020; Terreni et al., 2021). AMR is already a complicated issue, and its rise poses a serious threat to existing techniques for treating infectious diseases caused by bacteria. MDR bacteria are becoming increasingly common as they are able to tolerate many antibiotic classes (Bassetti et al., 2019). This raises doubt about the efficacy of established treatment approaches and typically correlates to higher death rates, longer hospital stays, and more expensive health care (Nelson et al., 2021). The word “superbugs” refers to bacterial strains that have developed broad resistance mechanisms, thus becoming resistant to the vast majority of currently available antibiotics, while Pan-drug-resistance (PDR) is a term, which is used if a bacterium is resistant to all antimicrobial agents. Sobouti et al. (2020) revealed that burnt children developed infections caused by PDR strains. Out of 115 clinical *A. baumannii* strains that were isolated from burnt children in Tehran, Iran, 36 (58%), 17 (27.5%), and 09 (14.5%) were identified as MDR, XDR, and PDR, respectively.

To date, there is no published study that compares the genetic differences and case reports of MDR and XDR strains reported in Saudi Arabia, Middle East, Central European and Asian countries. The purpose of this study is to present a comparative analysis of different MDR and XDR case reports from various countries of the world and Saudi Arabia. In addition, this study is designed to present the genetic differences in genes responsible for resistant mechanisms among MDR and XDR bacterial strains reported within Saudi Arabia and rest of the world. This study outlines the emergence of Superbug, XDR, and MDR bacterial strains, which is a major threat to public health. Several MDR and XDR case reports are discussed in detail, with a special emphasis on Saudi Arabian origins. Furthermore, this study highlights growing concerns for MDR and XDR strains throughout the globe. The findings are extremely important since they will aid in ensuring global health security, focusing efforts on antibiotic stewardship, and encouraging innovative research into novel antimicrobial drugs.

## 2 Driving factors for antibiotic resistance

One of the primary factors contributing to AMR is the widespread misuse and overuse of antibiotics. Antibiotics are routinely given in medical settings for viral illnesses or in situations where they might not be the best line of treatment (Tian et al., 2021). Most patients do not finish the prescribed antibiotic courses, unintentionally contributing to the emergence of resistance. In addition to using antibiotics for medicinal purposes, the agricultural sector has made contributions by stimulating the development and growth of animals and other non-therapeutic uses of antibiotics (Dankar et al., 2022; Walia et al., 2023). The etiology of antibiotic resistance is developed by multiple factors, each having their additional role. These include poor regulations and imprecision, accessibility to low and standard antibiotics, lack of awareness in healthcare facilities and overuse of antibiotic agents in poultry, agriculture and livestock to promote growth (Kariuki et al., 2023). Chiefly, the main factor responsible for the emergence of AMR is evolution, as warned by Sir Alexander Fleming (Iskandar et al., 2022; Caneschi et al., 2023).

Inappropriate prescription of antibiotics also leads to emergence of resistant bacterial strains (Giacomini et al., 2021). Identifying the pathogen responsible for a specific infection is a serious problem. According to a study conducted in the United States, it showed that the pathogen was only 7.6% identified in a total number of 17,435 hospitalized patients with community acquired pneumonia in comparison to Sweden, where molecular diagnostic procedures were used and resulting in an identification rate for the strain of 89% (Mohsin and Amin, 2023). Mostly antibiotics used in ICUs are inappropriate, unnecessary or suboptimal level (Ventola and therapeutics, 2015). There are multiple factors, which are associated with the over and inappropriate prescription of antimicrobial agents like lack of experience among physicians, diagnostic problems, and patient attendant influences on prescriber decisions. Improve the approach toward prescribing antibiotics would lead to a decrease in antibiotic resistance and ultimately improve patient overall health (Baraka et al., 2021). The extensive use of antimicrobial drugs in several sectors such as healthcare and agriculture, has led to the rapid emergence of bacterial resistance, thus decreasing the efficacy of traditional therapies (Ahmad et al., 2021).

In developed countries like Bangladesh, Pakistan and India, hospital regulations are poor and the overuse of antibiotics in poultry animals is unjust (Naher et al., 2020). The extensive usage of antimicrobial in farm and poultry animals as growth promoters is also another cause for transmission of resistance from animals to humans. There is also a lack of quality research in novel antibiotics production which is affecting both developing and developed countries (Aslam et al., 2018). Antimicrobials are used in aquaculture which are present at sub therapeutic level in water for a longer period of time resulting in AMR infections if considered in the long run (Santos and Ramos, 2018). Excessive use of antibiotics for COVID-19 led to more complications like treating chest infections, TB (tuberculosis), and typhoid infections (Mendelson et al., 2022). In nursing homes, a significant number of systemic antibiotics are used for prophylactic purposes against bacterial infections. This leads to serious adverse effects and the emergence of AMR (Sloane et al., 2020). In veterinary medicines the use of antimicrobials are very extensive for prophylactic purposes (Odoi et al., 2021). There has been an increase in the over-use of antibiotics because of patient treatment preferences and pressure on physician, which leads to over-prescription. There is a huge increase in the use of antibiotics in recent times and spreading quickly and more commonly (Ramachandran et al., 2019). The AMR has a direct relation with the consumption of antibiotics. For example, in respiratory tract infections where the prescription of these drugs is unnecessary (Roope et al., 2018).

The introduction of fake and contaminated medications with little therapeutic effect to the market is one such cause of AMR (Zabala et al., 2022). Due to the bacteria’s tendency to develop resistance at low drug concentration levels, it further worsens major problems in treating infection. Since the bacteria become resistant to the antimicrobials, these low medication concentration levels frequently result in therapeutic failures (Nicoloff et al., 2019). A study carried out in Cameroon in 2004 discovered that 32, 10, and 13% of a sample of 284 antimicrobial medications from 132 vendors were determined to be counterfeit versions of quinine, sulfadoxine/pyrimethamine, and chloroquine, respectively (Basco, 2004). Concern regarding counterfeit drugs continues to grow. Specifically, the public’s health is at risk from counterfeit antimicrobial drugs, which can have serious negative consequences for patients; rising morbidity and mortality as well as the development of drug resistance (Tabernero et al., 2019). The regulatory authorities are unable to impose rigorous laws limiting the “Over counter” sale of prescription medications in many low- and middle-income countries (LMICs) (Saleem et al., 2020). Despite government regulations prohibiting the sale of antimicrobials without a prescription and requiring that medication be dispensed under the supervision of a registered pharmacist exclusively, a study carried out in Tanzania found that 80–85% of people were overusing and inappropriately consuming pharmaceuticals (Poyongo and Sangeda, 2020).

A lack of funding, uncontrolled antibiotic manufacture and distribution, restricted access to high-quality, reasonably priced medications, and other poverty-related issues all play a role in the emergence of antibiotic resistance in underdeveloped nations. Poor patient compliance also contributes to the development of antibiotic resistance in the short term when treating acute infections, as well as in the long term when treating chronic diseases like tuberculosis. Notably, those experiencing extreme poverty are compelled to seek the assistance of dishonest doctors and drug dealers who provide counterfeit and outdated pharmaceuticals or low-trait antimicrobial dosages. So, the fact that people in poverty self-medicate is a major factor in their lack of access to medical care (Sharma et al., 2022). Poor healthcare infrastructures, limited access to high-quality services, and a lack of diagnostic capacity are some of the factors contributing to antibiotic overuse in LMICs (Sulis et al., 2023).

## 3 Global implications of antimicrobial resistance

**Figure 2:**
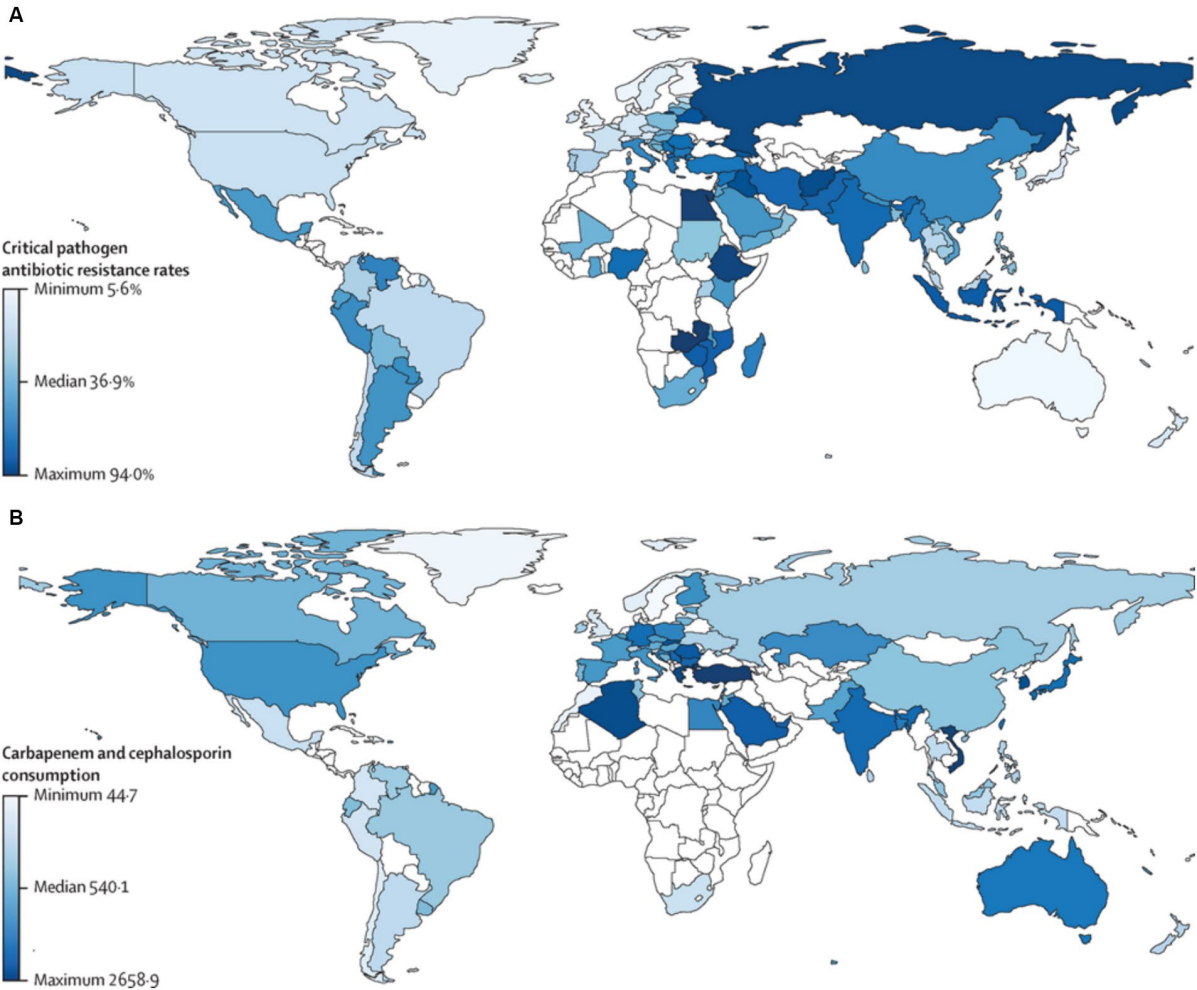
Critical pathogen antibiotic resistance rates and carbapenem and cephalosporin consumption by country. **(A)** Antibiotic resistance rate in humans for the critical pathogens in humans (96 observations). **(B)** Antibiotic consumption (in DDDs) in humans for carbapenems and cephalosporins (73 observations). Countries in white represent those with missing data. Pearson’s correlation between antibiotic resistance and consumption in humans was 0.30 (*p* = 0.021). DDD, defined daily doses per 1,000 individuals. Reprinted from Figure 1 in “Global antimicrobial-resistance drivers: an ecological country-level study at the human–animal interface” by Allel et al., (2023), licensed under Creative Commons Attribution (CC BY) license (https://creativecommons.org/licenses/by/4.0/).

Antimicrobial resistance is a major public health issue in the 21st century, posing a threat to the effective treatment of infections (Aljeldah, 2022). In 2019 alone, AMR is estimated to have contributed to 4.95 million deaths, with 1.3 million specifically caused by resistant infections (Tang et al., 2023). While AMR affects all countries worldwide, it poses a particularly significant concern for low- and middle-income countries (LMICs) (Hou et al., 2023). Projections suggested that by 2050, the global GDP might decrease by 1% per year, with developing countries facing a loss of 5–7%, translating to a staggering ultimate outcome ranging from $100 to $210 trillion. By 2050, multidrug-resistant tuberculosis alone could cost mankind $16.7 trillion (Fong, 2023). The Centers for Disease Control and Prevention (CDC) has estimated that AMR costs the US $55 billion annually, with $20 billion allocated to higher direct healthcare expenses and up to $35 billion contributing to additional societal expenditures due to lost productivity (Bergmann et al., 2022). AMR is a serious threat for all countries around the world, but it is of greater concern to be taken into consideration specifically in low and middle-income countries (Figure 2; Hou et al., 2023).

The excessive use of antibiotics during the COVID-19 pandemic has led to more complications, such as treating chest infections, tuberculosis (TB), and typhoid infections, which nowadays result in multidrug resistance, particularly in countries like India, Russia, and the Philippines (Dadgostar, 2019). Moreover, India and China are recognized as the biggest hotspots for AMR, while Brazil and Kenya are emerging places for AMR (Van Boeckel et al., 2019). The serious effects of AMR, including increased mortality and morbidity, higher healthcare costs, limited treatment options, and an imminent threat to contemporary medicine, go far beyond the realm of healthcare (Salam et al., 2023). It also has a significant negative economic impact, increases concern about global security, disrupts agriculture and ecosystems, and has an adverse effect on food safety and biodiversity (Broom et al., 2023). The infection and mortality rates of AMR are continually observed in light of the rapid growth of diseases (Stanley et al., 2022). According to estimates, 65,162 cases of AMR infection were diagnosed in the United Kingdom in 2019, compared to 61,946 cases in 2018 (Tang et al., 2023). Comparatively speaking, the European Center for Disease Prevention and Control (ECDC) estimated that the yearly AMR infection rate in the EU alone had surpassed 670,000 cases. About 1.27 million of the 4.95 million deaths attributed to bacterial AMR in 2019 were directly brought on AMR, according to data analysis from a previous study (Kakoullis et al., 2021; Gaur et al., 2022).

Multidrug resistant bacterial pathogens are common, as evidenced by a number of studies, particularly in food and ready-to-eat products. For instance, Badawy et al. (2022) conducted a study to find *S. aureus*-resistant strains isolated from cattle, buffalo, their environment, milk, and dairy products. They also looked at the extent to which MDR strains have contaminated livestock, the environment, and food. *Streptococcus aureus* (SA) was found in 17.9, 17.6, and 16.7% of the samples taken from cows, buffalo, and Karish cheese, respectively, according to the study’s findings. The *mecA* gene was present in roughly 19% of isolated SA strains. The isolates showed resistance to cefotaxime (73.1%), kanamycin (97%), nalidixic acid (86.6%), clindamycin (100%), and sulphamethazole-trimethoprim (65.6%). In the meanwhile, ciprofloxacin, amikacin, imipenem, and both cefoxitin and gentamycin were found to be effective against 95.5, 94, 86.6, and 77.7% of SA strains, respectively (Badawy et al., 2022). Furthermore, a study by Elbehiry et al. (2022) assessed the frequency of isolates of Pseudomonas in 320 specimens of chicken meat. Using Microflex LT, protein analysis was done on cultivated isolates. Pseudomonas isolates’ resistance was identified using Vitek® 2 AST-GN83 cards. In total, 69 samples were recognized to be contaminated by *Pseudomonas* species, comprising 18 isolates of *P. lundensis*, 16 isolates of *P. fragi*, 13 isolates of *P. oryzihabitans*, 10 isolates of *P. stutzeri*, five isolates of *P. fluorescens*, four isolates of *P. putida*, and three isolates of *P. aeruginosa*. Microflex LT properly identified all isolates of *Pseudomonas* (100%) with a score value of >2.00. The observed isolates were positively differentiated into multiple groups using PCA. 81.16% of the isolates of *Pseudomonas* were resistant to nitrofurantoin, 71% to ampicillin and ampicillin/sulbactam, 65.22% to cefuroxime and ceftriaxone, 55% to aztreonam, and 49.28% to ciprofloxacin when tested against antimicrobials. Elbehiry et al. (2022) reported that the susceptibilities for cefotaxime, ceftazidime, cefepime, and each piperacillin/tazobactam were 100, 98.55, 94.20, and 91.3%, respectively. A number of researchers conducted similar nature studies. For instance, a study by Algammal et al. (2022a), found MDR *Bacillus cereus* strains in fish meat. Furthermore, another study revealed that strains of *Aeromonas veronii* were found in fish. Through the use of PCR, it was discovered that the isolates carried the virulence genes for *act*, *ahp*, *ompAII*, *nuc*, *alt*, and *ser* with 100, 82.9, 61.7, 55.3, 44.7, 36.17, and 29.8% prevalence, respectively. The study showed that *blaTEM*, *blaCTX-M*, *blaSHV*, *tetA*, *aadA1*, and s*ul1* resistance genes were present in 29.8% (14/47) of the recovered *A. veronii* strains, which were also XDR to nine antimicrobial classes. Similarly, *blaTEM*, *blaCTX-M*, *blaSHV*, *tetA*, *aadA1*, and *sul1* genes were present in 19.1% (9/47) of the collected *A. veronii* strains that were MDR to eight classes (Algammal et al., 2022b).

## 4 Case reports

In this section, MDR and XDR case studies were collected from already published data. The data collection process was conducted randomly, without a predetermined bias toward any specific bacterial species. However, the primary objective was to investigate a detailed study, which has mentioned a story about the patient condition, previous antibiotic usage, genetic basis of the MDR and XDR strain, and its susceptibility and resistance patterns. The MDR and XDR case reports are classified on the basis of previously published guidelines presented by Magiorakos et al. (2012).

### 4.1 XDR carbapenemase-producing hypervirulent *Klebsiella pneumoniae* strain in Taiwan

*Klebsiella pneumoniae* is one of the well-known opportunistic pathogen in the Enterobacteriaceae family, which is frequently associated with infections such as urinary tract infections, pneumonia, surgical site infections, and bloodstream infections. The bacteria may colonize human mucosal surfaces and the gastrointestinal tract, making it highly transmissible in healthcare settings. Individuals with compromised immune systems, including those in hospitals, patients receiving prolonged antibiotic therapy, and those suffering from chronic illnesses, are at a higher risk of acquiring *K. pneumoniae* infections (Wang et al., 2020; Wyres et al., 2020).

#### 4.1.1 Case presentation

A study by Huang and colleagues conducted a study at the Taipei Veterans General Hospital in Taiwan. *K. pneumoniae* strains that produced carbapenemase (KPC) were subsequently isolated from clinical specimens. These strains were used for pulsed-field gel electrophoresis (PFGE) and multilocus sequence typing (MLST), as well as for an analysis of the capsule types and the presence of *rmpA*/*rmpA2*. After being tested in an *in vivo* mouse lethality study to confirm its virulence, the strain positive for *rmpA*/*rmpA2* underwent whole-genome sequencing (WGS) to identify its genetic characteristics (Huang et al., 2018).

#### 4.1.2 Findings

During the study period, a total of 63 KPC-producing *K. pneumoniae* strains were identified in the hospital. The *ST11* and capsular genotype *K47* were the two groups of *K. pneumoniae* strains that produced KPC-2, out of a total of 62 strains identified. Among these, *blaKPC-2* was present in the majority (*n* = 62), while *blaKPC-3* was present in just one strain. *ST11* and capsular genotype *K47* were shared by all isolates carrying KPC-2. The strain that carried the *rmpA* and *rmpA2* genes was limited to *TVGHCRE225*. According to the string test, this strain likewise exhibited the hypermucoviscous phenotype. This strain was isolated from an intra-abdominal abscess in an 83-year-old woman. A fistula that was situated between the colon’s splenic flexure and the spleen was the source of the intra-abdominal abscess. The abscess was drained from the patient using a catheter guided by percutaneous ultrasonography. *ESBL*-phenotype *K. pneumoniae*, *E. coli*, and *Enterococcus faecium* were detected in the initial pus culture. Despite receiving tigecycline and ceftazidime, the patient’s condition worsened. Vitek 2 System evaluation of the second pus culture from the drainage tube collected 10 days later revealed the establishment of a carbapenem-resistant *K. pneumoniae* strain (*TVGHCRE225*) with imipenem MIC ≥16 mg/L. This strain was likewise susceptible to amikacin (MIC ≤2 mg/L) and demonstrated resistance to fluoroquinolones and cephalosporins. Six days after this strain was obtained, the patient died from septic shock despite receiving amikacin. *TVGHCRE225* is an XDR strain based on its MICs for tigecycline and colistin, which were 8 and 4 mg/L, respectively. The study concluded that as there is an immediate risk to human health, active surveillance concentrating on KPC hypervirulent *K. pneumoniae* strains is required (Huang et al., 2018).

### 4.2 XDR *Pseudomonas aeruginosa* (urinary tract infection)

#### 4.2.1 Case presentation

*Pseudomonas aeruginosa* belongs to the class of Gammaproteobacteria. XDR *P. aeruginosa* (XDR-PA) infections can have a high fatality rate, be extremely dangerous, and highly challenging to treat (Ahani Azari and Fozouni, 2020). Mendes Pedro et al. (2024) carried out a retrospective study on the patient records. The goals of the study were to describe the XDR-PA population, outcomes, and microbiological features found in a Portuguese university hospital center. Resistance to carbapenems, aminoglycosides, third- and fourth-generation cephalosporins, piperacillin-tazobactam, and fluoroquinolones constituted the definition of XDR-PA (Mendes Pedro et al., 2024).

#### 4.2.2 Findings

XDR *P. aeruginosa* identification was more frequent in male patients with an average age of 64.3 ± 17.5 years. Of the tested isolates, 71.5% were sensitive to ceftazidime-avibactam (CZA). Ceftolozane-tazobactam (C/T) demonstrated susceptibility in 77.5% of the isolates. Except for cystic fibrosis, patients with confirmed infections were only eligible for antibiotic regimens with XDR-PA coverage. Colistin (41 cases), CZA (39 cases), and C/T (16 cases) were the most commonly given antibiotics. Of the total 130 patients, 35.1% of infected patients died; those with hematologic malignancy had a significantly higher death rate (50.0%, *p* < 0.05), followed by those with bacteremia (44.4%, *p* < 0.05) and those taking colistin (39.0%, *p* < 0.05), especially those with respiratory infections (60.0%). One non-susceptibility to colistin case was also reported (Mendes Pedro et al., 2024).

### 4.3 MDR and XDR case from Pakistan

#### 4.3.1 Case presentation

Zakir et al. (2021) conducted a study at a local hospital in Lahore (Pakistan) to ascertain the prevalence of MDR and XDR *S. Typhi*. For the purpose of identifying the bacteria, blood samples (*n* = 3,000) were collected. In the results, 600 positive cultures were detected, with strains of XDR *S. Typhi* (46.1%) and *MDR S. Typhi* (24.5%) constituting the greater part of these cultures. Males (60.67%) were more likely than females (39.33%) to contract a disease caused by resistant *Salmonella* strains, with children aged 0–10 (70.4%) suffering the most from it (Zakir et al., 2021).

#### 4.3.2 CST findings

**Figure 3:**
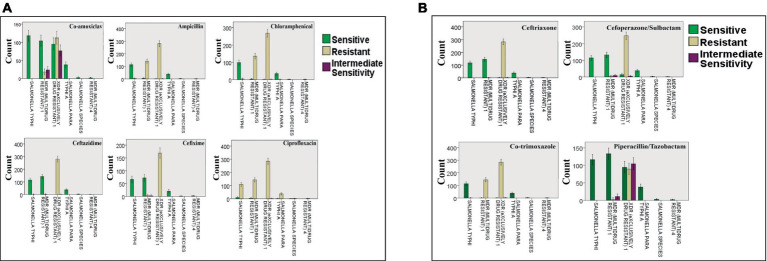
Resistance of *Salmonella* strains against: **(A)** co-amoxiclav, ampicillin, chloramphenicol, ceftazidime, cefixime, and ciprofloxacin; **(B)** ceftriaxone, cefoperazone/sulbactam, co-trimoxazole, and piperacillin/tazobactam. The bars present the number of cases with resistant, sensitive, and intermediately sensitive strains against the mentioned antibiotics. Reprinted from Figures 2 and 3 in “Emerging trends of multidrug-resistant (MDR) and extensively drug-resistant (XDR) *Salmonella typhi* in a tertiary care hospital of Lahore, Pakistan” by Zakir et al. (2021), licensed under Creative Commons Attribution (CC BY) license (https://creativecommons.org/licenses/by/4.0/).

A shockingly high prevalence of XDR *S. Typhi* cases (48.24%) was seen in both the outpatient department (OPD) and general ward, with MDR *S. Typhi* cases (25.04%) ranking second. Based on the statistical analysis, it was found that neither patient gender nor age had an impact on the incidence of resistance in MDR and XDR *S. Typhi* strains. Similar resistance strains were discovered to be present in several wards as well as in hospitalized and outpatient department patients against the four tested antibiotics, namely; ciprofloxacin, imipenem, meropenem, and azithromycin. Furthermore, chloramphenicol and ampicillin indicated maximum resistance in the pediatric ward and outpatient department. Antibiotics with the most efficacy were found to be piperacillin/tazobactam and co-amoxiclav. Figure 3 deficits the resistance pattern exhibited by *Salmonella* strains as reported by Zakir et al. (2021).

### 4.4 MDR case from China

#### 4.4.1 Case presentation

Isoniazid (INH) and rifampin (RIF) are the prominent first-line anti-tubercular drugs. Luo et al. sequenced particular genes of *Mycobacterium tuberculosis* that are linked to resistance to RIF and INH in 157 phenotypic MDR isolates in order to ascertain the prevalence and distribution of mycobacterial mutations in these genes. Among the strains, it was demonstrated that the Beijing genotype predominated (84.1%). Additionally, 93.6% of strains had changes in the 81 bp core area of *rpoB*, and 88.5% of phenotypic MDR isolates exhibited mutations in the promoter of *inhA*, the intergenic region of *oxyR-ahpC*, and a structural gene (*katG*). The Beijing and non-Beijing genotypes, as well as the clustered and non-clustered strains, did not significantly differ in terms of codon mutations (Luo et al., 2019).

#### 4.4.2 CST findings

The most common mutations affecting RIF and INH were *Ser315Thr* in *KatG* (56.1%) and *Ser531Leu* in *rpoB* (55.4%), respectively. Between MDR-TB and other drug-resistant tuberculosis (DR-TB), there was little variation in RIF and INH resistance. The results indicated that 127 were single drug-resistant (RIF), 123 were double drug-resistant (RIF + INH), 98 were triple drug-resistant (RIF + INH + EMB), and four were quadruple drug-resistant (RIF + INH + SM + EMB). Farmers made up 94 of 195 DR-TB patients (48.2%), 239 of 711 DS-TB patients (33.6%), and 91 of 157 MDR-TB patients (58.0%). Lung cavitation’s were more common in MDR-TB patients (28/157 [17.8%]) than in DS-TB patients (54/711 [7.6%]) (Luo et al., 2019).

### 4.5 MDR case from India

#### 4.5.1 Case presentation

A major global issue is the recent rise of hypervirulent, MDR *K. pneumoniae* that cause severe infections and high fatality rates. We still lack answers to the problems these hypermucoviscous *K. pneumoniae* strains pose in terms of the best ways to treat, manage, and regulate them. A group of hypervirulent and XDR *K. pneumoniae* ST5235 isolates that were resistant to carbapenems and polymyxins and were causing newborn sepsis at an Indian tertiary care hospital were investigated by Banerjee et al. Nine isolates of *K. pneumoniae* from neonatal sepsis were examined in terms of their clinical significance, antimicrobial susceptibility profile, presence of ESBL production, and genes responsible for carbapenemases (classes A, B, and D) and aminoglycoside resistance. The study examined a series of fatal cases of newborn sepsis at a tertiary-care hospital in India that were caused by *hvKP* and had widespread drug resistance to carbapenems and polymyxins (Banerjee et al., 2021).

#### 4.5.2 CST findings

This analysis highlighted the challenges that these emerging pathogens represent in underdeveloped nations. The majority of the newborns had respiratory distress (66%), were preterm/premature (55.5%), and had very low birth weights (<1,500 g, 66%). Pre-eclampsia, heart disease, and irregular vaginal discharges were among the problems experienced by all but one (88.8%) mother of these cases of newborn sepsis during or before delivery. Lower-segment Cesarean sections (LSCS) accounted for the mode of delivery in the majority of instances (66.6%). Imipenem/meropenem, vancomycin, and piperacillin-tazobactam were the empirical treatments used on all the cases. Treatment with polymyxin B was also administered as a last resort. Nevertheless, all of the newborns eventually passed away from the disease. The culture profile is given in Table 1 (Banerjee et al., 2021).

**Table 1:** Resistance profile of *Klebsiella pneumoniae* isolates reported by Banerjee et al. (2021).

Antimicrobial resistance profile																
Isolates	AMC	PTZ	CXM	CRO	CFP	CPM	LVX	ETP	IPM	MEM	AMK	GEN	CEP	SXT	CL^*^	PB^*^
BK1	R	R	R	R	R	R	R	R	R	R	I	R	R	S	R	R
BK2	R	R	R	R	R	R	R	R	R	R	R	R	R	R	R	R
BK3	R	R	R	R	R	R	R	R	R	R	R	R	R	R	R	R
BK4	R	R	R	R	R	R	R	R	R	R	I	R	R	S	R	R
BK5	R	R	R	R	R	R	R	R	R	R	R	R	R	R	R	R
BK6	R	R	R	R	R	R	R	R	R	R	R	R	R	R	R	R
BK7	R	R	R	R	R	R	R	R	R	R	R	R	R	R	R	R
BK9	R	R	R	R	R	R	R	R	R	R	R	R	R	R	R	R
BK14	R	R	R	R	R	R	R	R	R	R	R	R	R	R	R	R

AMC, Amoxicillin/clavulanic; PTZ, Piperacillin/tazobactam; CXM, Cefuroxime; CRO, Ceftriaxone; CFP, Cefoperazone; CPM, Cefipime; LVX, Levofloxacin; ETP, Ertapenem; IPM, Imipenem; MEM, Meropenem; AMK, Amikacin; GEN, Gentamicin; CEP, Ciprofloxacin; SXT, Trimethoprim/sulfamethoxazole; CL, Colistin; PB, Polymyxin B; R, Resistant; I, Intermediate; S, Susceptible. ^*^Microbroth dilution method. Reprinted from Table 2 in “Extensively drug-resistant hypervirulent Klebsiella pneumoniae from a series of neonatal sepsis in a tertiary care hospital, India” by Banerjee et al. (2021), licensed under Creative Commons Attribution (CC BY) license (https://creativecommons.org/licenses/by/4.0/).

### 4.6 MDR case from Egypt

#### 4.6.1 Colistin resistant *Klebsiella pneumoniae* and *Escherichia coli* strains

##### 4.6.1.1 Case presentation

Zafer et al. (2019) reported MDR case *K. pneumoniae* and *E. coli* strains from Egypt. Hospitalized cancer patients provided samples for the collection of 450 enterobacterial isolates, including 234 *K. pneumoniae*, 200 *E. coli*, and 16 *Enterobacter*. Before isolating the colistin-resistant Enterobacterial isolate, 440 patients had received antibiotics.

##### 4.6.1.2 CST findings

Resistance to ampicillin/sulbactam (84%), levofloxacin (73%), piperacillin/tazobactam (72%), ciprofloxacin (71%), meropenem (53%), and tobramycin (52%) was less common compared to resistance to cefazolin (92%), ceftriaxone (91%), ceftazidime (89%), and cefepime (86%), and trimethoprim/sulfamethoxazole (85%). Forty isolates (8.8%) showed colistin resistance when evaluated by broth microdilution, of which 18 (45%) showed meropenem resistance. In the same way, E-tests revealed that 8% of the isolates were resistant to colistin. Ultimately, a VITEK 2 system study revealed that 31%, developed extended-spectrum beta-lactamases. 16 of the 40 colistin-resistant isolates were found to have carbapenemases by PCR screening for the most prevalent ones; of these, 9/40 were positive for *blaOXA-48* and 7/40 were positive for *blaNDM* (Zafer et al., 2019).

### 4.7 MDR cases from Kingdom of Saudi Arabia

#### 4.7.1 *Acinetobacter* spp., *Enterobacter* spp., *Escherichia coli*, *Klebsiella pneumoniae*, *Proteus* spp., and *Pseudomonas aeruginosa*

##### 4.7.1.1 Case presentation

Al Hamdan et al. (2022) conducted a study in Riyadh, Saudi Arabia to evaluate the emerging issue of MDR and XDR bacterial strains. According to their findings, a greater rate of resistance in the isolated gram-negative bacterial strains in 2017 (68.2%) compared to 2018, with 969 (64.3%) of the 1,508 gram-negative bacterial strains was noticed (Table 2). Patients in the 70–79 age range had the highest rate of MDR-gram-negative bacterial strains (77%), followed by those in the 90+ age range (75%). There was no apparent variance in resistance between male and female patients.

**Table 2:** The rate of antibiotics susceptibility among MDR-GNB isolates collected in 2017 and 2018, by Al Hamdan et al. (2022).

% Susceptibility																	
Species	Year	AMIK	AMP	AMC/CLV	CZO	FEB	CAZ	CRO	CXM	CIP	LEVO	GENT	IMP	MRP	NFN	PIP/TAZ	TRIMETH
*E. coli*	2017	91	6	22	11	43		35	34	70	40	87	99	99	51	40	49
	2018	71	24	53	50	52		50	49	62	84	97	96	61	63	72	47
*Klebsiella* spp.	2017	84		35	35	48		44	43	82	75	76	99	99	30	68	61
	2018	96		16	45	43		42	42	78	90	97	97	79	21	56	60
*Proteus* spp.	2017	79	4	34	36	51		45	41	93	87	64	62	62		42	47
	2018	93	29	28	25	45*		39	30	89	68	61	30	89		36	59
*Enterobacter* spp.	2017	71	0	0	0	29		21	0	93	93	70	100	100		29	0
	2018	94	0	0	0	56		41	0	91	88	85	74	91		47	71
*Acinetobacter* spp.	2017	42				10	3			14	16	29	13	13		6	19
	2018	82				59	59			76	82	71	71	76		47	88
*Pseudomonas* spp.	2017	81				29	29			79	90	76	48	47		29	
	2018	96				50	49			85	94	67	70	85		39	

%Susceptibility rate = Number of susceptible isolates/Total number of isolates × 100. Boxes in green, yellow, and red color reflect susceptibility rate of: 90–100, 70–89, and <70%, respectively. AMIK, Amikacin; AMP, Ampicillin; AMC/CLAV, Amoxicillin/clavulanic acid; CZO, Cefazolin; FEB, Cefepime; CAZ, Ceftazidime; CRO, Ceftriaxone; CXM, Cefuroxime; CIP, Ciprofloxacin; LEVO, Levofloxacin; GENT, Gentamicin; IMP, Mipenem; MRP, Meropenem; NFN, Nitrofurantoin; TAZ, Piperacillin/tazobactam; and TRIMETH, Trimethoprim. Reprinted from Table 1 in “Evaluating the prevalence and the risk factors of Gram-negative multi-drug resistant bacteria in eastern Saudi Arabia” by Al Hamdan et al. (2022), licensed under Creative Commons Attribution (CC BY NC) license (https://creativecommons.org/licenses/by/4.0/).

##### 4.7.1.2 CST findings

The isolates of gram-negative bacteria with high MDR were found in samples taken from INP units. *Proteus* spp. (72.4%) and *Enterobacter* spp. (86.5%) had the highest rates of resistance throughout the course of the 2 years. In Gram-negative bacteria (GNB) isolated from patients utilizing a central line (≥72 h) (81%), multidrug resistance was most common (7%), in GNB isolated from patients utilizing a mechanical ventilator (≥72 h) and patients who had previously had surgery within 4 weeks of the beginning of GNBs (75%). To sum up, the study revealed a significant prevalence of MDR-GNBs, with a 64.3% overall rate. A major direct risk factor for the development of MDR-GNBs has been shown to be the use of a mechanical ventilator for three or more calendar days. As a result, we need to highlight and support hospital antimicrobial stewardship efforts. It is imperative that healthcare personnel possess knowledge about MDR-GNBs and prevention strategies. Increasing awareness among the public and medical professionals about the appropriate use of antibiotics will aid in preventing the acquisition of MDR strains (Al Hamdan et al., 2022).

#### 4.7.2 Extended-spectrum beta lactamase producing *Escherichia coli*

##### 4.7.2.1 Case presentation

The primary objective of the Abalkhail et al. (2022) study was to determine the prevalence and pattern of ESBL-*E. coli* antibiotic resistance in patients with urinary tract infections in Riyadh, Saudi Arabia. A total of 2,250 urine samples from patients suffering from urinary tract infections (UTIs) were collected at King Fahd Medical City in Riyadh, Saudi Arabia. Standard biochemical procedures were then used to culture and identify the microbial species found in the samples. The identification of ESBL-producing strains of *E. coli* was done using a double-disk synergy test; the resistance of these strains to antimicrobial drugs was evaluated using an *in vitro* approach and the Clinical Laboratory Standard Institute (CLSI) criteria.

##### 4.7.2.2 CST findings

Of the 1,523 *E. coli* isolates, 33.49% had ESBL-*E. coli*; 67.27% of these isolates came from women and 33.7% from men. Patients under the age of 50 accounted for 55% of the total ESBL-*E. coli* isolates identified. Although most of the ESBL-*E. coli* isolates were sensitive to various carbapenems (imipenem, meropenem, and ertapenem), aminoglycosides (amikacin), and nitrofurantoins, nearly all of the isolates were resistant to cephalosporins (ceftriaxone, cefotaxime, cefepime, cefuroxime, and cephalothin) and penicillin (ampicillin). The most common bacterium associated with UTIs, *E. coli*, has developed antibiotic resistance. This is an important factor in deciding the best course of antibiotic therapy (Abalkhail et al., 2022).

#### 4.7.3 Carbapenem-resistant *Klebsiella pneumoniae*

##### 4.7.3.1 Case presentation

A total of 191 CRKP isolates were collected and retrieved from clinical specimens of patients at King Fahad Armed Forces Hospital in Jeddah, Saudi Arabia, during the study by Alhazmi et al. (2022). All isolates that exhibited resistance or intermediate susceptibility to imipenem, meropenem, or ertapenem, three carbapenem-based antimicrobials, were classified as appropriate for the study.

##### 4.7.3.2 CST findings

**Figure 4:**
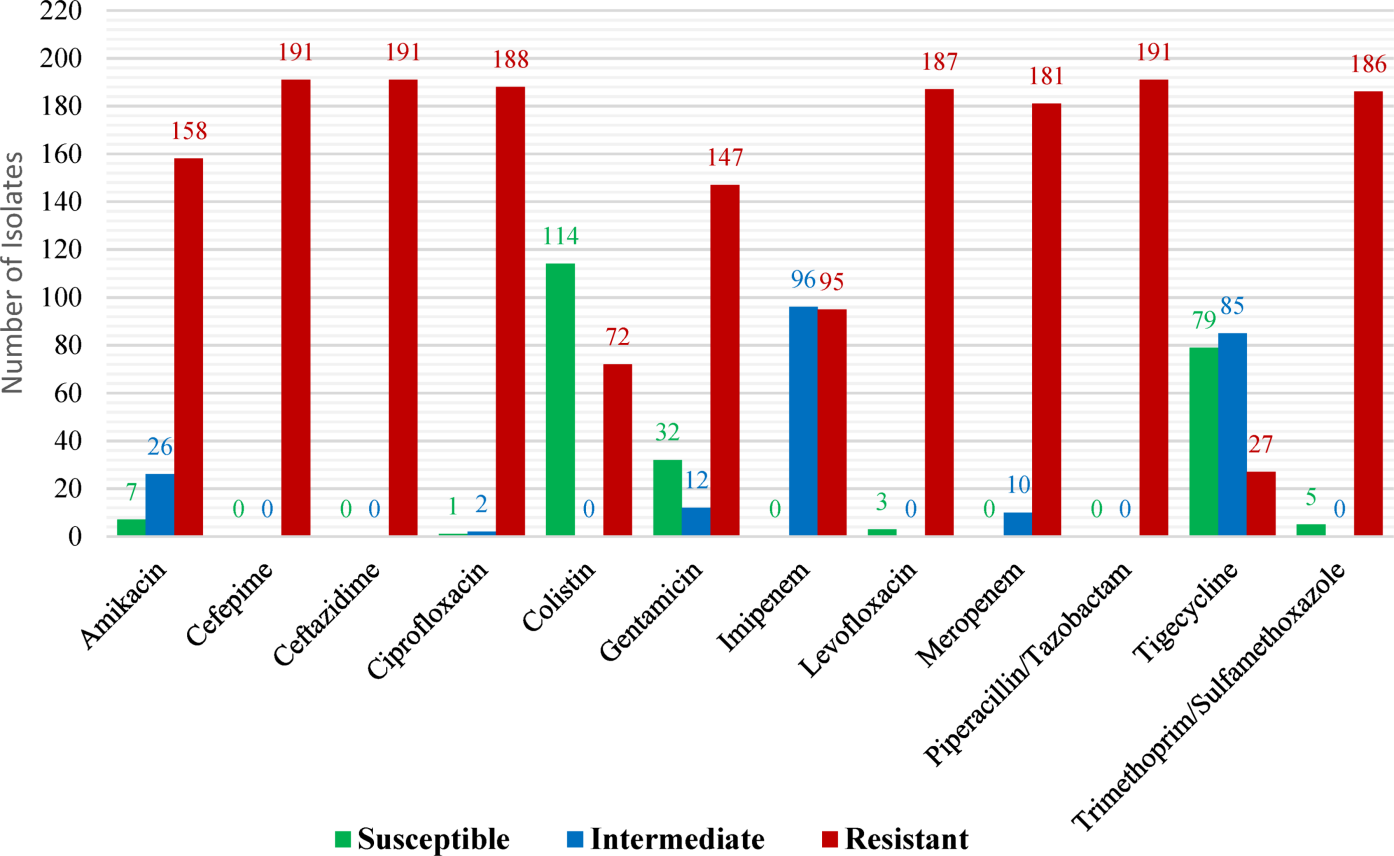
Antimicrobial profiles of carbapenem-resistant *Klebsiella pneumoniae* isolates as reported by Alhazmi et al. (2022). Reprinted from Figure 1 in “The molecular characterization of nosocomial carbapenem-resistant Klebsiella pneumoniae co-Harboring bla_NDM_ and bla_OXA-48_ in Jeddah” by Alhazmi et al. (2022), licensed under Creative Commons Attribution (CC BY) license (https://creativecommons.org/licenses/by/4.0/).

While low (14%) and considerable (37.7%) levels of resistance were detected against tigecycline and colistin, respectively, all CRKP demonstrated resistance to ceftazidime, cefepime, and piperacillin/tazobactam. The most often found carbapenemase gene (82%) was *blaOXA-48*, with *blaNDM* being found in 27 (14%) isolates. *OXA-48* may have become endemic in Saudi Arabian hospitals, as evidenced by the high prevalence of the pathogen among *K. pneumoniae* isolates found in the current study (Figure 4). The current study’s significant *OXA-48* prevalence among *K. pneumoniae* isolates could indicate that *OXA-48* has become endemic in Saudi Arabian hospitals. Because of their high level of carbapenem resistance, these isolates pose a threat to healthcare communities, including patients and healthcare personnel. These findings imply that in order to track and stop the spread of any multidrug-resistant (MDR) bacteria, including CRKP, appropriate surveillance strategies and the application of molecular diagnostic techniques are necessary (Alhazmi et al., 2022).

#### 4.7.4 *Escherichia coli*, *Klebsiella pneumoniae*, and *Enterobacter* spp.

##### 4.7.4.1 Case presentation

A retrospective analysis was carried out from 2015 to 2018 in various ICUs of a Saudi Arabian tertiary-care hospital. No PDR Enterobacteriaceae cultures were found among the 227 *Enterobacteriaceae* cultures that were part of this study; of those, 60% were either MDR or XDR.

##### 4.7.4.2 CST findings

The predominant bacterial strain found in MDR/XDR cultures were *E. coli* (51.4%), *K. pneumoniae* (33%), and *Enterobacter* spp. (19%). Furthermore, carbapenems (47%) and cephalosporins (21.3%) were the three most often used antibiotic classes, followed by piperacillin/tazobactam (53%). Merely 61% patients were deemed appropriate to receive antibiotic therapy. Drug-resistant *Enterobacteriaceae* infections were found to be highly prevalent in the study and were linked to a high death rate. In 40% of the instances, a microbiological cure was obtained, and 84% of patients died while they were hospitalized. The average length of hospital stay was 27 days, which can be directly linked to MDR (Alkofide et al., 2020).

## 5 The race against antibiotic scarcity: time for immediate intervention

**Figure 5:**
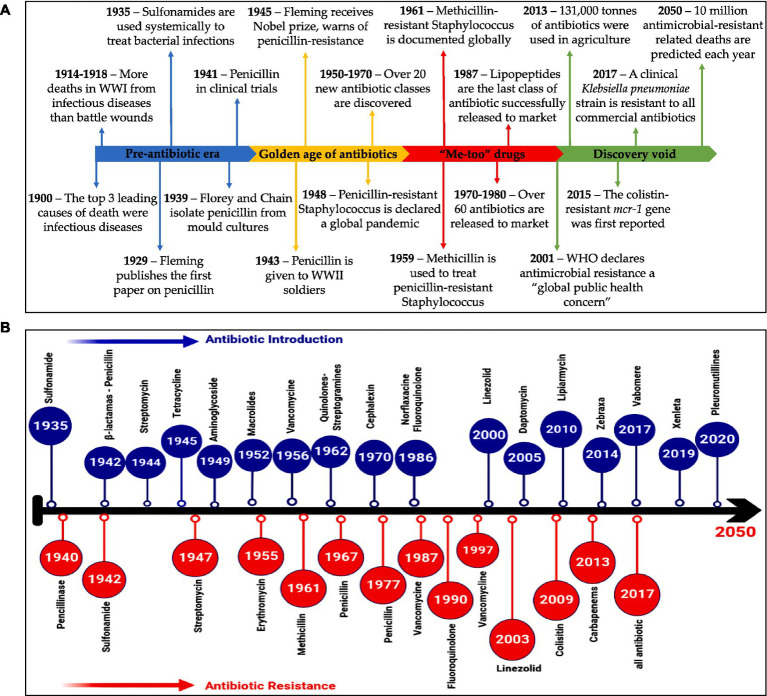
**(A)** A summary of events in the AMR timeline (Browne et al., 2020). **(B)** Timeline illustrates antibiotics evolution (Helmy et al., 2023). **(A)** Reprinted from Figure 1 in “A new era of antibiotics: the clinical potential of antimicrobial peptides” by Browne et al. (2020), licensed under Creative Commons Attribution (CC BY) license (https://creativecommons.org/licenses/by/4.0/). **(B)** Reprinted from Figure 1 in “Antimicrobial resistance and recent alternatives to antibiotics for the control of bacterial pathogens with an emphasis on foodborne pathogens” by Helmy et al. (2023), licensed under Creative Commons Attribution (CC BY) license (https://creativecommons.org/licenses/by/4.0/).

As the peak of the AMR threat emerges, experts have declared that we are getting closer to the age beyond which there will be no more effective antibiotics (Chandler, 2019). The development and discovery of new antibiotics have slowed significantly since the late 1990s; in the last 30 years, the FDA has approved only three new antibiotics. There is a lack of a sufficient pathway for antibiotics supply and usage, which may compromise international efforts to control drug-resistant diseases, according to two recent WHO assessments on clinical and preclinical drug development (Terreni et al., 2021). There are 252 lead compounds in the preclinical phase of drug development that are in the very early phases of testing and may become available in approximately 10 years. Among the 50 antibiotics in clinical studies for drug discovery, the majority offer limited benefits (Butler et al., 2023). WHO has issued a new warning about the worldwide threat of AMR due to the lack of new antibiotic discoveries and the withdrawal of certain large pharmaceutical companies from the antimicrobial domain (Dhingra et al., 2020). Major pharmaceutical companies engaged in AMR research have pulled out of the market owing to a “lack of incentives” and declining profits, which is further worsening the threat. According to two recent WHO surveys, small and medium-sized enterprises are primarily responsible for driving clinical and preclinical drug research (Anderson et al., 2023). Furthermore, in comparison to the more substantial and active preclinical biotechnology pipeline, the overall amount of spent on research and development in clinical development is insufficient to meet global health demands. As a result, the pharmaceutical industry’s R&D stream is running down of innovative drugs (Figure 5) (Mittal et al., 2023).

## 6 Recent treatment regimens

### 6.1 Phytochemicals

Plant-derived compounds have a broad range of structural variations and are found in various plant components, including the roots, leaves, bark, flowers, and seeds. They are secondary products of plant metabolism. For many years, drug development focused more on plant-derived chemicals to improve the biological activity of already-approved antibiotics or as a possible source of novel antimicrobial agents that are effective against a variety of diseases, including MDR and XDR bacterial strains (Jubair et al., 2021).

In literature, a number of studies are conducted employing the antibacterial properties of berberine against MDR bacterial pathogens. Berberine appears to be a unique type inhibitor of the MexXY-dependent aminoglycoside efflux in *P. aeruginosa*, according to a research by Morita et al. (2016). The antibacterial properties of methanolic extracts from *Phellodendri Cortex* or *Coptidis Rhizoma* were investigated by the researchers. Berberine was the most prevalent benzylisoquinoline alkaloid in the two extracts. The study’s findings indicate that in MDR *P. aeruginosa* strains, the methanolic extracts significantly decreased resistance to amikacin (aminoglycosides). By requiring the MexXY multidrug efflux system, berberine decreased *P. aeruginosa’s* resistance to aminoglycosides. Furthermore, berberine also decreased the minimum inhibitory concentrations (MICs) of aminoglycosides in *Achromobacter xylosoxidans* and *Burkholderia cepacia*, two species that possess intrinsic multidrug efflux systems that are extremely comparable to the MexXY. Additionally, employing a pseudomonad deficient of the other four main Mex pumps, berberine suppressed MexXY-dependent antibiotic resistance of additional classes, including as cephalosporins (cefipime), macrolides (erythromycin), and lincosamides (lincomycin). Even though the well-known efflux inhibitor phenylalanine-arginine beta-naphthylamide (PAβN) antagonized aminoglycoside in a MexXY-dependent way, *P. aeruginosa’s* amikacin resistance was reduced by berberine at a lower dose when PAβN was present. In addition, berberine increased the synergistic effects of amikacin and piperacillin in isolates of MDR *P. aeruginosa* (Morita et al., 2016). MDR *Acinetobacter baumannii* strains are a serious concern to the world health care system because they can cause severe infections in intensive care units and are quickly becoming resistant to the existing antibiotics. Because of its consistent therapeutic efficacy, berberine hydrochloride (BBH) derived from Berberis and has been used extensively as an antibacterial agent. It was shown that BBH and antibiotics had synergistic effects against MDR *A. baumannii in vitro*. The antibacterial activity of BBH alone was shown to be poor (MIC≥256 mg/L). However, it significantly enhanced MDR bacteria’ sensitivity to antibiotics with FICI values <0.5 and even reversed their antibiotic resistance (to antibiotics like ciprofloxacin, tigecycline, sulbactam, and meropenem, among others). Following this, an *in vivo* study revealed that BBH with sulbactam had greater antibacterial effectiveness than monotherapy in a neutropenic mouse thigh infection model. Additionally, the BBH’s antibiotic-sensitizing mode of action was assessed. Less absorption of BBH due to its binding to the AdeB transporter protein and enhancement of *adeB* gene expression may result in less antibiotic extrusion by the AdeABC pump. In MDR strains, knockout of the *adeB* gene resulted in higher absorption of BBH, decreased antibiotic sensitization, and reduced synergistic effects between antibiotics and BBH. When combined, BBH and antibiotic resistance efficiently re-sensitize this MDR pathogen to a variety of antibiotics that are now barely effective, suggesting that BBH might be a good therapeutic adjuvant candidate to battle MDR *A. Baumannii* (Li et al., 2021).

In terms of antibacterial activity, isolates of *S. aureus* with MIC 1.9 mg/L were significantly affected by sanguinarine, which was isolated from the root and aerial sections of *Chelidonium majus L*. This antibacterial effect is attributed to the iminium bond, methoxy substitution, and charge on the quaternary nitrogen atom (Cushnie et al., 2014). According to a study, sanguinarine inhibits the production of cytokinetic Z rings in *E. coli*, hence preventing bacterial cell proliferation. Another study found that this alkaloid effectively inhibits MRSA growth by weakening the bacterial cytoplasmic membrane. MRSA will release autolytic enzymes to cause cell lysis when it comes into contact with sanguinarine (Qi et al., 2024). Another study by Zhang et al., found that sanguinarine had strong inhibitory effects (MIC 7.8 μg/mL) on isolates of *Providenica rettgeri* resistant infection. Results from field emission scanning electron microscopy (FESEM), confocal laser scanning microscopy (CLSM), and crystal violet staining indicated that sanguinarine inhibited the formation of bacterial biofilms and decreased the intracellular ATP content (Zhang et al., 2020).

The alkaloids tetrahydrosecamine (piperidine) and streptanol (indole) that were extracted from *Rhazya stricta Decne* showed notable antibacterial action against MRSA, *E. coli*, and *P. aeruginosa* by rupturing the bacterial cell membrane (Khan et al., 2016). Similarly, dihydrocheleryhtrine and N-methylcanadine, two isoquinoline alkaloids that were first isolated from the root bark of Zanthoxulum tingoassuiba, demonstrated strong antibacterial action (MIC 60 μg/mL) against MRSA (Kuete et al., 2008). *Carica papaya* leaf extract, *Datura stramonium* leaf extract, and *Piper nigrum* fruit extract was examined for their antibacterial activity against Gram-positive and Gram-negative bacteria, such as MDR *S. aureus*, *P. aeruginosa*, *A. baumannii*, and *E. coli*. Better effectiveness against Gram-positive bacteria was demonstrated by all extracts. The *Piper nigrum* extract, however, showed no action against MDR. *Carica papaya L.* and *Datura stramonium L*. ethanolic extracts showed a greater zone of inhibition than methanol extracts. Alkaloids, steroids, glycosides, phenols, saponin, and carotenoids were found in the phytochemical screening of both plants, with tannin being found exclusively in Datura extract (Costa et al., 2017). The Southeast Asian plant *Piper sarmentosum Roxb*. has been found to contain a number of alkaloids; these include trimethoxycinnamic acid, piplartine, and langkamide, which are found in the roots and stems of the plant. A number of amide alkaloid compounds have also been extracted from the plant’s leaves. The leaves’ methanolic extracts demonstrated strong antibacterial action against MDR pathogens, including *E. coli* and MRSA, with MICs of 100 mg/mL (Ismaili et al., 2017).

### 6.2 Essential oil

Essential oils (EOs) have an important role in the control of MDR and XDR. For instance, a recently published study by Coșeriu et al. evaluated the antimicrobial activity of 16 common EOs on MDR *P. aeruginosa* clinical isolates, including the determination of the effects on *mex* efflux pumps gene expression. After the disk diffusion screening, the MIC of the EO that shown antibacterial activity was further evaluated. Although cinnamon EO had the strongest antibacterial action, real-time RT-PCR was utilized to assess its impact on the gene expression of the *mex A*, *B, C, E*, and *X* efflux pumps. All strains of *P. aeruginosa* were suppressed by cinnamon EO, which was followed by thyme EO (37.5%, *n* = 27) and lavender EO (12.5%, *n* = 9). Less effective were the other EOs. All MDR *P. aeruginosa* isolates were suppressed by cinnamon at a dose of 0.05% v/v, according to the MIC detection. The essential oils of lavender, clove, peppermint, basil, thyme, and turmeric showed varying outcomes; most of them exhibited activity at concentrations greater than 12.5% v/v. *MexA* and *mexB* (66.5%) were frequently underexpressed, according to research on the effects of cinnamon EO on *mex* efflux pumps. With 100% antimicrobial activity against clinical isolates of *P. aeruginosa* that are MDR, XDR, and PDR, cinnamon EO has demonstrated remarkable results at very low concentrations. These results, coupled with a significant alteration of the RNA messaging system, support the oil’s potential for use as an adjuvant treatment that could impact therapeutic outcomes (Coșeriu et al., 2023). An additional study was conducted to figure out the antibacterial effects of *Cinnamomum zeylanicum* bark essential oil (CZ-EO) against the tested XDR clinical isolates of vancomycin-resistant *E. faecium* (VR *E. faecium*), *A. baumannii*, *P. aeruginosa*, and *E. coli*. The XDR isolates were found to be highly sensitive to CZ-EO. Following MRSA (the most sensitive isolate) were VR *E. faecium*, *E. coli*, *P. aeruginosa*, and *A. baumannii*. The MRSA and VR *E. faecium* MIC value ranges were 0.15–1.25, 0.15–2.5, 0.15–5, and 0.31–10 μL/mL, respectively. For CZ-EO, the corresponding values for MIC50, MIC90, MBC50, and MBC90 parameters were 1.25, 5, 2.5, and 5 μL/mL, respectively (Saki et al., 2020). The EO of black zira demonstrated both bactericidal and bacteriostatic properties against multiple pathogens, such as *P. aeruginosa* and *E. coli*. The MBC values of the oils ranged from 1 to 16 mg/mL, whereas the MIC values varied from 1 to 8 mg/mL. Alkaloids, flavonoids, saponins, tannins, and phenols were found via phytochemical examination (Mabhiza et al., 2016).

### 6.3 Phage therapy

Bacteriophages, often known as phages, are viruses that enter bacterial cells and multiply. In the case of lytic phages, this results in the death of host cells. For more than a century, phage treatment has been utilized to treat bacterial infections; however, due to the introduction of antibiotics, the restricted efficacy of certain phage strains, and lack of knowledge about phage therapies, the therapy was largely replaced by antibiotics (Broncano-Lavado et al., 2021). Because of their capacity to avoid harming normal flora due to their specificity, biofilm degradation mechanisms, and ability to evade typical antibiotic resistance mechanisms, phages have recently attracted attention as a viable treatment alternative in the era of antimicrobial resistance (Xu et al., 2021). Nowadays, phage therapy is mostly used in Eastern and Western Europe. Numerous case reports have confirmed the efficacy of phage therapy in treating MDR and XDR bacterial infections in both humans and animals (Forti et al., 2018; Jeon and Yong, 2019). For instance, Lin et al. (2021) attempted to study the synergistic activity of phage *PEV20*-ciprofloxacin combination powder formulation. After infecting mice with *P. aeruginosa*, animals were given either freshly spray-dried single *PEV20* (106 PFU/mg), single ciprofloxacin (0.33 mg/mg), or a combination of *PEV20* and ciprofloxacin treatment using a dry powder insufflator. This allowed researchers to examine the synergistic efficacy of *PEV20* and ciprofloxacin. After 24 h of therapy, lung tissues were taken for flow cytometry analysis and colony counting. The combination powder of *PEV20* and ciprofloxacin significantly decreased the clinical *P. aeruginosa* strain’s bacterial burden in mice lungs by 5.9 log10 (*p* < 0.005). When *PEV20* or ciprofloxacin alone was administered to the animals, no apparent reduction in the bacterial population was seen. Evaluation of the lungs’ immune responses revealed a correlation between the bactericidal action of the *PEV20*-ciprofloxacin powder and decreased inflammation (Lin et al., 2021).

Nir-Paz et al. reported a case of polymicrobial infection. After being admitted to the trauma unit, a 42-year-old male with a right distal femoral fracture and a bicondylar tibial plateau fracture had an XDR *A. baumannii* and an MDR *K. pneumoniae* isolated from his left tibia on day nine. After the patient had treatment for 6 weeks with piperacillin/tazobactam and 8 weeks with meropenem and colistin, the XDR *AbKT722* strain was identified and shown resistance to both colistin and carbapenem. Over the course of 5 days, meropenem and colistin were combined with phages *ΦAbKT21phi3* and *ΔKpKT21phi1* (which target *A. baumannii* and *K. pneumoniae*, respectively) and given intravenously (5 × 107 PFU/mL). After a week, the patient received a second dose of phage therapy on 6th day, and showed indications of improvement. At last, after 8-month follow-up no bacterial strains were isolated from the patient (Nir-Paz et al., 2019). Wu et al. documented the case of four patients undergoing phage therapy as a considerate measure to treat lung carbapenem-resistant *A. baumannii* infections in the China COVID-19-specific intensive care unit (ICU). Phage-resistant strains emerged after Patient 1 got *ΦAb124* phage by nebulization, altering the second course of treatment, which involved combining *ΠAb121* with *ΦAb124* to produce a cocktail. The other patients received the cocktail—not single phages—from the start of their treatment to prevent resistance because *in vitro* research demonstrated that the combined phages could reduce the recurrence of resistant bacteria. Patient 2’s jugular incision healed after the cocktail was administered. Patients 1 and 2’s chest radiographs improved enough that they were released from the hospital at the completion of their treatment. Patient 3’s carbapenem-resistant *K. pneumoniae* infection proved deadly, and 10 days after starting phage therapy, the patient died from respiratory failure. Following a week of phage treatment, Patient 4 showed improvement and was allowed to leave the ICU. However, a month later, the patient passed away from respiratory failure (Wu et al., 2021).

### 6.4 Vaccination

Targeting MDR and XDR bacterial strains with vaccines can reduce AMR in a number of ways, such as (i) lowering the frequency of infections brought on by these microbes, (ii) lowering the amount of antibiotics used to treat infections brought on by both susceptible and resistant strains, (iii) preventing infection spread to unvaccinated individuals, and (iv) preventing the spread of resistance genes to susceptible strains of both pathogenic and non-pathogenic species (López-Siles et al., 2021). Formalin-inactivated entire *ATCC19606* type strain cells were used in one of the earliest studies describing the preclinical development of an *A. baumannii* vaccine. This work showed that intramuscular immunization with these cells produced serum antibodies, including both IgG and IgM, against a variety of outer membrane antigens. In a mouse model of acute sepsis, mice were protected against infection by both the vaccine strain and MDR clinical isolates. Additionally, passive immunization against the formalin-inactivated cells provided protection (McConnell and Pachón, 2010). According to García-Quintanilla et al., immunization with inactivated cells derived from a lipopolysaccharide (LPS)-deficient mutant similarly induced a strong antibody response and offered notable defense against clinical isolates of *A. baumannii* (García-Quintanilla et al., 2014). In two cases, vaccination of mice models of infection with live, attenuated strains of *A. baumannii* has been described. After two doses, a live, attenuate vaccine lacking in thioredoxin produced significant antibody levels and showed a 100-fold reduction in mouse pathogenicity (Ainsworth et al., 2017). In an additional study, sera from mice inoculated with the vaccine showed opsonophagocytosis activity against *A. baumannii* strains *in vitro*, and immunization with *E. coli* OMVs expressing the *A. baumannii* outer membrane protein Omp22 produced antigen-specific IgG. Significantly, mice that had received the *Omp22* expressing OMVs as part of their immunization showed considerable protection against infection when inoculated against it via passive immunization with antisera (Huang et al., 2016).

Although vaccination works efficiently to prevent bacterial infections, it has not been shown to be effective against infections brought on by MDR and XDR strains. Typically, vaccinations only stimulate the immune system against the intended pathogen, reducing the possibility of negative consequences on the human microbiota. In certain situations, identifying the antigens expressed by disease-causing or antibiotic-resistant strains but not by strains that are part of the commensal microbiota may make it feasible to develop vaccines that only target these strains (Lipsitch and Siber, 2016).

### 6.5 Implementation of preventive measures

Despite several recommendations over the years, infection control programs (ICPs) and antimicrobial stewardship programs have been implemented in several countries to promote the appropriate use of antimicrobials and prevent MDR and XDR infections. The interventions vary widely by region and effect (Apisarnthanarak et al., 2013). A few studies have revealed how education for ICPs, hand hygiene campaigns, and the judicious use of carbapenem may decrease the incidence of XDR and MDR infections (Cheon et al., 2016). However, more and more studies have shown that the drug resistance of AB has generally increased; the prevalence of CRAB has increased worldwide, and numerous hospital outbreaks in ICUs have been reported (Teerawattanapong et al., 2017). Some studies have even suggested enhanced stewardship programs are urgently needed and have been developed to avoid the spread and potential outbreaks by MDRAB, especially in high-resistance endemic settings (Molter et al., 2016). Environmental cleaning is an important component of a comprehensive strategy to control healthcare-associated infections, especially in wards such as the ICU, where patients are compromised (Wilson et al., 2016). One study showed that comprehensive measures with environmental cleaning in a NICU environment were effective and significantly reduced the incidence rate of methicillin-resistant *S. aureus* (Li et al., 2017).

One of the main tactics in the fight against antimicrobial resistance is antibiotic stewardship, which is the effort to optimize the usage of antibiotics in clinical practice. To assist the implementation of antimicrobial and diagnostic stewardship throughout the spectrum of health care, the CDC Office of Antibiotic Stewardship release updated guidelines and resources (Palmer et al., 2021). The CDC keeps track of data on antibiotic use and stewardship implementation in human healthcare settings in order to assess advancements and guarantee that all patients have equitable access to high-quality medical care (Noval et al., 2023). In order to prevent the misuse of antibiotics, the CDC established four fundamental components of outpatient antimicrobial stewardship: a dedication from all members of the healthcare team to participate in antibiotic stewardship; action for policy and practice that can be converted into quantifiable results; monitoring and reporting of antibiotic use to direct modifications and monitor advancements; and knowledge and experience from experts who can help with resource and instructional material creation. The CDC issued additional guidelines, focusing on “high-priority conditions,” which frequently impact the general public in the outpatient context and present chances for effective stewardship interventions. These consist of skin and soft tissue infections (SSTIs), urinary tract infections (UTIs), and acute respiratory infections (ARIs) (Marcelin et al., 2020).

## 7 Challenges in addressing AMR and prevalence of MDR/XDR and superbugs

Obviously one of the biggest threats to global health is AMR, which continues to evolve faster than the discovery of novel antibiotics. A variety of issues with widespread consequences include the challenge in combating antibiotic resistance and the frequency of bacterial strains that are MDR, XDR, and superbugs (Dehbashi et al., 2020). The evolution of resistant strains is largely caused by the overuse and misuse of antibiotics, which gets worse with self-medication, insufficient treatment regimens, and improper prescribing practices (Nabaweesi et al., 2021). Furthermore, delayed targeted treatment and the spread of resistant variations are caused by empirical therapies, the inability to quickly identify specific bacterial strains, and the lack of point-of-care tests as well as rapid diagnostics (Singh et al., 2021; Ryu et al., 2023). Biofilm infections continue to be a major concern in healthcare services because they are extremely resistant to antibiotics. The isolation of quorum-sensing compounds, limiting the propagation of biofilms (Tamfu et al., 2022), utilization of cell based drug delivery systems (Kamal et al., 2022), and combination approaches are among the few creative and successful antibiofilm strategies (Hawas et al., 2022). The aforementioned antibiofilm techniques are still in early stages and further clinical trials are required to make them available for clinical practice.

The necessity for international cooperation and uniform surveillance is highlighted by the challenge of preventing the cross-border spread of MDR, XDR, and superbugs caused by the interconnection of healthcare systems worldwide (Bedenić et al., 2023). Additionally, the limited supply of available therapies is made worse by the slow rate of new antibiotic discovery, which leaves a major gap in our ability to counteract the spread of developing resistance (Sulis et al., 2022). There are still many misconceptions regarding antibiotics that contribute to non-compliance and the continuation of resistance; therefore, raising public awareness and educating the public about antibiotics remains a major task (Nahar et al., 2020). To address these concerns and limit the rising threat of AMR and the prevalence of resistant bacterial strains, an effective plan encompassing improved diagnostics, deeper monitoring, appropriate antibiotic intake, and international collaboration is required.

## 8 Future prospective for management of MDR and XDR infection/strains

Although there are many areas on this list and they can be difficult to follow, putting these ideas into practice is essential to the ongoing global control of AMR (Medina and Pieper, 2016). Enhancing diagnostic and prescribing procedures, lowering the use of antibiotics in agriculture, creating new antimicrobials, antimicrobial stewardship initiatives, providing more equal access to drugs, and enhancing surveillance and infection control protocols are all significant fundamental concepts for managing AMR and the emergence of MDR/XDR strains in the future (Bassetti and Garau, 2021; Hetta et al., 2023).

The advent of novel diagnostic techniques is one of the most anticipated breakthroughs in the management of MDR/XDR strains and infections (Muthukrishnan, 2021). The necessity to modify empirical treatment to local epidemiology, patient risk stratification, and stewardship measures is highlighted by regional variations in the rates of resistance (Al Salman et al., 2021). Currently, empirical treatment is still the most often used method; nevertheless, it also plays a role in antibiotic misuse and subsequent transmission of AMR. In order to effectively focus therapies and, when feasible, quickly switch from broad-spectrum antibiotics, prompt diagnoses are essential (Soeroto et al., 2019). Moreover, pure cultures are needed and time-consuming classical growth-based methods of determining antibiotic sensitivity are employed (Tjandra et al., 2022). Non-purified samples, however, can be experimented with using innovative diagnostic approaches based on nucleic acid amplification, nucleic acid hybridization, or immunodiagnostic procedures (Mauk et al., 2018). Fast point-of-care diagnostics and antibiotic susceptibility testing are among the expected outcomes of such methods. It is anticipated that this will shorten the duration of treatment and facilitate the transition to evidence-based care, preventing the overuse of antibiotics and the development of AMR (Godman et al., 2021).

There is an urgent need for antimicrobial stewardship programs. Primary care physicians and stewardship teams working together, as well as treatment algorithms for optimal antibiotic dose and de-escalation based on susceptibility and culture results, are all essential components of antimicrobial stewardship programs (Donà et al., 2020; Hwang and Kwon, 2021). In order to encourage the discovery and development of novel antimicrobial drugs that could aid in the fight against MDR infections, the government must also provide financial incentives to researchers (Mantravadi et al., 2019). Specifically, comparative studies between new agents and existing antibiotics are required to determine the role of new agents in treatment approaches against MDR/XDR infections (Kanj et al., 2022). The comparatively limited number of patients with specific MDR illnesses makes it difficult to conduct randomized controlled studies with the necessary number of patients in a timely manner (Horcajada et al., 2019; Losito et al., 2022).

In recent years, advances in nanotechnology have been made in the treatment and prevention of MDR/XDR infection and biofilm formation (Mba and Nweze, 2021). The development of therapies based on nanomaterials holds promise for treating bacterial infections that are challenging to treat, as they may be able to overcome existing pathways associated with acquired drug resistance (Khorsandi et al., 2021). Furthermore, because of their unique size and physical makeup, nanoparticles can target biofilms and treat infections that are resistant to conventional antibiotics (Eduardo Pérez-Martínez and Zenteno-Cuevas, 2020). Finally, we recommend additional research to seek long-term solutions for MDR and XDR infection prevention and management.

## 9 Conclusion

In conclusion, this study was an attempt to present an in-depth evaluation of antimicrobial resistance in the form of MDR and XDR bacterial strains through case reports from various geographic locations, including Saudi Arabia, Taiwan, Poland, Pakistan, India, China, and Egypt, indicating the alarming global prevalence of resistant bacterial strains. The diverse resistance profiles of the detected strains make it increasingly difficult to apply conventional therapies, thus limiting the range of available antibiotic therapy. These events emphasize the critical need for innovative management and control strategies to combat these resistant strains. The emergence of MDR and XDR strains highlights the necessity for a thorough understanding of genetic changes and regional variances. Specifically, alerting trends, such as substantial resistance rates displayed by gram-negative bacterial strains in Saudi Arabia, underscore the severity of MDR and XDR scenarios.

Lastly, urgent action is required to minimize the impact of these resistant strains on public health. This action should encompass increased surveillance, appropriate utilization of antibiotics, and extensive research. Healthcare professionals, researchers, and governments must collaborate to successfully navigate the evolving MDR and XDR landscape, preserve effective therapies, and safeguard the efficacy of currently available antibiotics.

## Author contributions

BA: Conceptualization, Data curation, Formal analysis, Funding acquisition, Investigation, Methodology, Project administration, Resources, Software, Supervision, Validation, Visualization, Writing – original draft, Writing – review & editing.
